# Can birth weight predict offspring’s lung function in adult age? Evidence from two Swedish birth cohorts

**DOI:** 10.1186/s12931-022-02269-2

**Published:** 2022-12-15

**Authors:** Aleksandra Sakic, Magnus Ekström, Shantanu Sharma, Peter M. Nilsson

**Affiliations:** 1 Department of Clinical Sciences, Lund University, Skane University Hospital, Malmö, Sweden; 2 Department of Respiratory Medicine and Allergology, Lund University, Lund, Sweden; 3 Department of Clinical Sciences, and Department of Internal Medicine, Lund University, Skåne University Hospital, Jan Waldenströms Gata 15, 5th floor, 20502 Malmö, Sweden

**Keywords:** Birth weight, Cohort, Epidemiology, Gestational age, Lung function

## Abstract

**Background:**

Associations between birth weight (BW) and adult lung function have been inconsistent and limited to early adulthood. We aimed to study this association in two population-based cohorts and explore if BW, adjusted for gestational age, predicts adult lung function. We also tested adult lung function impairment according to the mis-match hypothesis—small babies growing big as adults.

**Methods:**

We included 3495 individuals (aged 46.4 ± 5.4 years) from the Malmo Preventive Project (MPP), Sweden, born between 1921 and 1949, and 1401 young to middle-aged individuals (aged 28.6 ± 6.7 years) from the Malmo Offspring Study (MOS) with complete data on BW and gestational age. Adult lung function (forced vital capacity [FVC], forced expiratory volume in one second [FEV1] and the FEV1/FVC-ratio) were analysed as level of impairment (z-score), using multiple linear and logistic regressions.

**Results:**

BW (z-score) did not predict adult lung function in MPP, whereas BW was a significant (p = 0.003) predictor of FEV1 following full adjustment in MOS. For every additional unit increase in BW, children were 0.77 (95% CI 0.65–0.92) times less likely to have impaired adult lung function (FEV1). Moreover, adults born with lower BW (< 3510 g) showed improved lung function (FEV1 and FEV1/FVC in MOS and MPP, respectively) if they achieved higher adult body weight.

**Conclusions:**

Adults born with lower birth weight, adjusted for gestational age, are more likely to have impaired lung function, seen in a younger birth cohort. Postnatal growth pattern may, however, compensate for low birth weight and contribute to better adult lung function.

**Supplementary Information:**

The online version contains supplementary material available at 10.1186/s12931-022-02269-2.

## Background

Growing evidence indicates that early life events and growth are important for later lung health irrespective of smoking habits, which in itself is a well-known risk factor affecting lung health [1–4]. A systematic review by Saad et al., based on literature through January 2015, has shown that low birth weight is associated with lower lung volumes in adulthood independently of other known birth-, childhood- or adult risk factors that may influence lung function [5].

According to the DOHaD (Developmental Origins of Health and Disease) concept, a fetus can undergo both adaptive and non-adaptive responses to changes in the intrauterine environment and thereby persistently alter physiological homeostasis that may influence the long-term risk of disease [6–8]. The predictive adaptive response (PAR) hypothesis suggests that a fetus in some way can predict conditions in the postnatal environment and thereby modify its physiological development, to optimize its survival in this environment, regardless of potentially harmful long-term health consequences. If there is a ‘mismatch’, that is, if the postnatal environment differs from the predicted, the long-term health consequences may be harmful. However, if the predicted environment matches the postnatal environment, offspring will be healthy. An example of an adaptive response is reduced fetal growth or low birth weight in order to adapt to a predicted scarce caloric postnatal environment. However, a non-adaptive response could lead to an increased risk of disease later in life even without mismatch. The non-adaptive response is pathophysiological and associated with, for instance, maternal obesity, gestational diabetes, or exposure to tobacco smoking [8].

Intrauterine growth restriction (IUGR) that can be caused by maternal, placental, fetal and/or environmental factors, may have a negative impact on lung development and adult lung function [1, 2, 9, 10]. Birth weight is often used as an indicator of fetal growth [8]. The predictive adaptive responses may occur across the entire range of birth weight, not only in low birth weight children [8]. IUGR is nowadays diagnosed by ultrasound [11] and thus hard to reproduce in historical cohorts. However, by adjusting birth weight for gestational age it is possible to investigate long-term health consequences of infants born small-for-gestational-age (SGA). Impaired lung function in adult life is strongly associated with increased risk of developing cardiovascular disease, type 2 diabetes and activity-related breathlessness, which in turn is associated with worse health status [12–14].

Data on birth weight and adult lung function are limited; studies are mostly restricted to adolescence and early adulthood [10, 15–17], or lack data on gestational age [5, 16, 18].

This observational study *aimed* to examine the association between birth weight, adjusted for gestational age, and lung function in adult life in two separate population-based projects in both men and women; the Malmo Preventive Project (MPP) in older and the Malmo Offspring Study (MOS) in younger subjects. Our hypothesis was that low birth weight or specific post-natal growth patterns could be associated with adult lung function.

## Methods

### Study populations

#### Malmo Preventive Project (MPP)

MPP is a large population-based cohort that started in 1974, inviting mostly middle-aged men and women residing in the city of Malmo, Sweden, with a participation rate of over 70% [19, 20]. A total number of 33,346 individuals (22,444 men born between 1921 and 1949, and 10,902 women born between 1926 and 1949) attended the initial health screening performed between 1974 and 1992. This included a physical examination, blood sampling, screening spirometry and a self-administered questionnaire. The questionnaire comprised 260 questions related to lifestyle habits, medical history and symptoms of disease, as well as social factors and family history. Smoking history was obtained through a questionnaire including several questions regarding smoking habits. We used only the first one *“Are you a smoker?”* (yes/no). Level of education was also obtained through a questionnaire. Questions were *“Have you completed elementary school (folkskola), primary school or similar?”, “Have you completed junior secondary school (realskola), vocational school or similar?”* and *“Have you completed secondary school, folk high-school (*= *adult education centre) or similar?”* with answer options *“yes”,* or *“no”.*

A total of 28,934 individuals underwent spirometry using a Spirotron apparatus (Drägerwerk AG, Lubeck, Germany), performed by specially trained nurses, including values for peak flow, forced vital capacity (FVC), and forced expiratory volume in one second (FEV_1_). The manoeuvre was performed with the individual in an upright standing position without a nose clip. More details on lung function testing and the measurement of pulmonary function have been previously published [13, 20–24]. Men were screened mostly between 1974 and 1982 and women between 1982 and 1992. Hence, the results were obtained during varying follow-up time and at different ages [19, 20].

Data concerning perinatal factors, including birth weight, birth length and gestational age (based on last period) were collected from local hospital archives in southern Sweden, available for 4359 individuals (3883 men and 476 women), derived from two nested case–control studies aimed to study risk on breast- or prostate cancer [25, 26]. For the present study, only participants in MPP with both birth data and spirometry were included (N = 3495), see Additional file 2: Fig. S1.

#### Malmo Offspring Study (MOS)

MOS is an ongoing population-based cohort since 2013 (attendance rate 47%) that invites adult children and grandchildren of participants in the Malmo Diet Cancer-Cardiovascular Arm (MDC-CV) study [27]. Participants were invited to undergo a baseline evaluation, including a questionnaire, physical examination and blood sampling. Participant’s height (meters) was measured with their legs together looking straight ahead in indoor clothing without shoes and caps. Weight (kg) was measured on a calibrated beam or digital scale. Body mass index (BMI, kg/m^2^) was calculated. Smoking history and level of education were obtained through a questionnaire. One of the questions regarding smoking was *“Do you smoke?”* with answer options *“yes, I smoke regularly”, “yes, I smoke occasionally”, “no, I have stopped”* or *“no, I have never smoked”*. Furthermore, participants were asked to tick the option that corresponds to the highest level of education they had completed.

Screening spirometry was performed by specially trained nurses using a MasterScope spirometer (JAEGER, Washington, US) with the individual standing with a nose clip providing values for FVC and FEV_1_. The spirometry maneuver was performed in accordance with guidelines from ATS/ERS [28]. The highest values of FVC and FEV_1_ were used.

Birth parameters for the study participants were obtained from the Swedish Medical Birth Register (MBR) starting in 1973. A personal 10-digit identity number, unique for every Swedish citizen, was used to link the MOS data collected at the health examination with the MBR data. Birth weight (gram) was available for 1401 (born 1973 or later) out of 3200 participants, see Additional file 2: Fig. S2. In addition, other birth parameters, such as gestational age and birth length were obtained from the MBR. Gestational age was calculated as the time period between the last day of the menstrual period of the mother and the date of offspring birth expressed in full weeks.

### Statistical analysis

Statistical analysis was performed using IBM SPSS statistics for windows version 25.0 (IBM Corp., Armonk, N.Y., USA). The average for all continuous variables was described as the mean and standard deviation (SD). The distribution of categorical variables was expressed as percentages. An independent-samples t-test was conducted to compare the means of continuous variables for men and women. A two-tailed p < 0.05 was taken to indicate statistical significance.

The predicted lung function values were calculated using The Global Lung Function Initiative (GLI) reference values [29]. Absolute lung function values (FEV_1_, FVC, FEV_1_/FVC-ratio) and birth weight were standardized as z-scores based on GLI [29] and Marsal et al. [11] normal reference values, respectively. In these calculations, sex, age, height and ethnicity are taken into account for lung function z-score; and sex and gestational age for birth weight z-score. The z-score indicates deviation from normal expressed as how many standard deviations a measured value is located from the predicted mean; 90% of healthy subjects will have z-scores values within ± 1.64. A threshold for clinically important lung function impairment was defined as a z-score less than the lower limit of normal (LLN), defined as < − 1.64 [30].

**Fig. 1:**
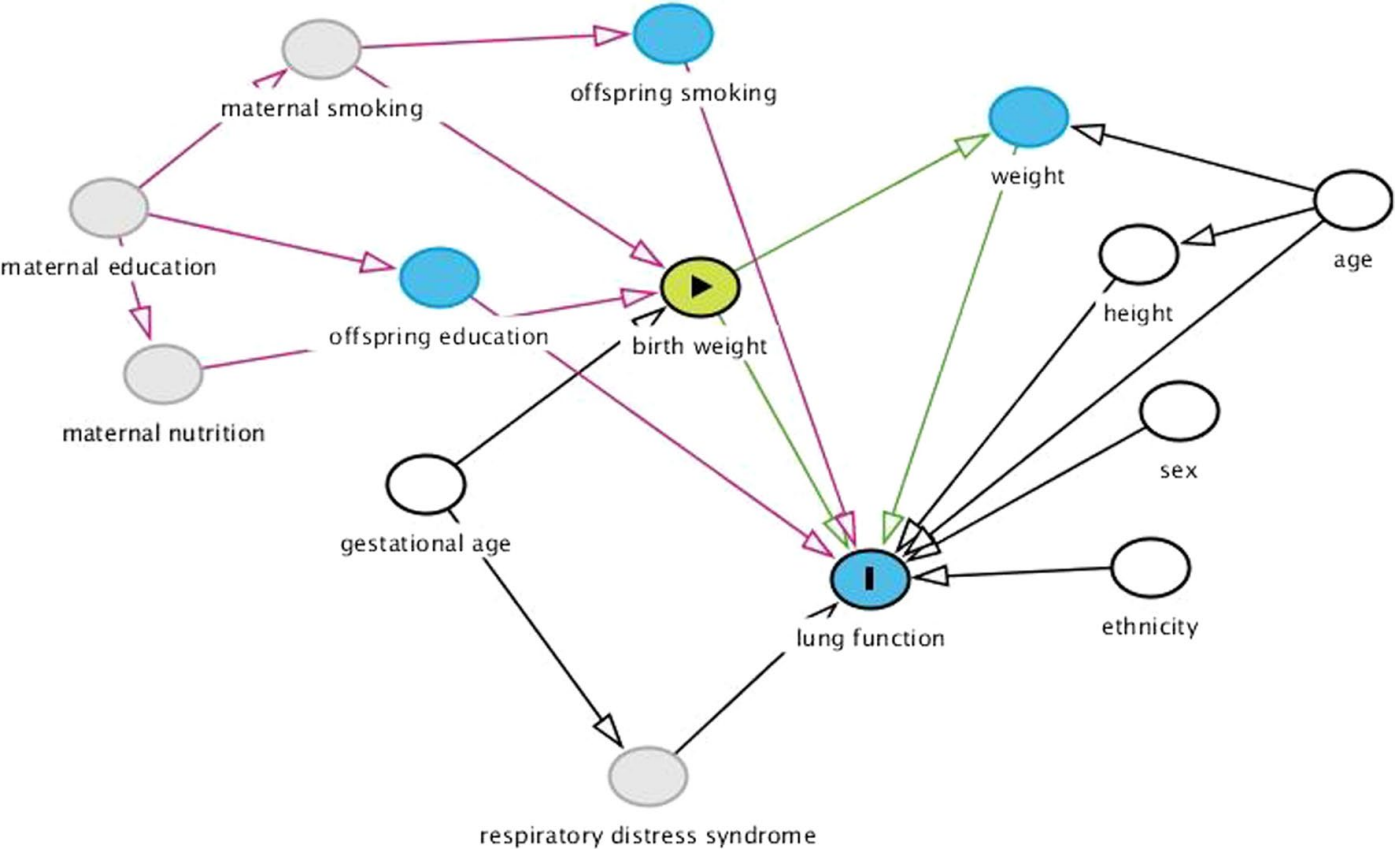
Directed Acyclical Graph (DAG) of the assumed causal relations between study variables

Covariates (confounders) to include in the models were selected based on the literature and current knowledge in the field using a causal diagram (DAGitty software) [31], shown in Fig. 1.

Selected covariates (confounders) to adjust for in the final models were offspring’s educational level and smoking status, whereas gestational age, sex, height, age and ethnicity are variables that have already been taken into account when calculating z-scores.

Linear regression models were conducted in a structured, step-wise approach:

*Firstly*, a univariate linear regression was computed to assess the association between each of the birth parameters (absolute birth weight, gestational age, birth weight expressed as z-score, birth length) and lung function in adult life (expressed as z-score) in the total population.

*Secondly*, hierarchical multiple regression and mixed linear model analysis accounting for clustering between siblings was used to assess the ability of birth weight deviations to predict lung function deviations in adult life using birth weight (z-score) as independent variable and FEV_1_, FVC and FEV_1_/FVC-ratio as continuous, dependent variables, respectively. First analysis was run unadjusted (Model 0). Offspring’s educational level was entered in *Model 1* while the offspring’s smoking status was added to *Model 2.*

Then, logistic regression was performed to calculate the odds ratio (OR) for having impaired lung function in adult life (z-score lung value less than − 1.64) using birth weight (z-score) as an independent variable and FEV_1_, FVC and FEV_1_/FVC-ratio as categorical dependent variable, respectively. Further adjustments for potential covariates were made in two models. In *Model 1,* adjustment was made for offspring’s educational level while in *Model 2,* adjustment for offspring’s smoking habits was added.

Furthermore, a two-way between-groups analysis of variance (ANOVA) was conducted to explore the impact of birth weight and achieved adult weight on lung function transformed to z-score. Birth weight (absolute value) and weight in adult life were divided into two categories: high and low, with medians as cut-offs: 3510 g (g) and 74 kg, respectively. Participants were then stratified into four groups (Group 1: lower birth weight in combination with lower adult weight; Group 2: higher birth weight and higher adult weight; Group 3: higher birth weight and lower adult weight; and Group 4: lower birth weight and higher adult weight).

## Results

The general characteristics of the study populations are presented for MPP (Table 1) and for MOS (Table 2) showing means ± SD and proportions presented for all subjects.

**Table 1:** Descriptive statistics among men and women from *Malmo Preventive Project (MPP)*

	Total population (N = 3495)	Men (N = 3330)	Women (N = 165)
Age (years)	46.4 ± 5.4	46.4 ± 5.3	49.4 ± 7.2
Birth characteristics			
Birth weight (gram)	3529.6 ± 547.2	3537.3 ± 546	3373.8 ± 549.6
Birth weight (z-score)	0.11 ± 2.9	0.13 ± 2.9	− 0.15 ± 1.3
Birth length (cm)	51.6 ± 2.5	51.6 ± 2.5	51.5 ± 2.9
Gestational age (days)	279 ± 16	279.3 ± 16.4	275.2 ± 13.8
Education n (%)			
Primary school	746 (21.5)	702 (43.2)	44 (39.6)
Secondary school	423 (12.2)	403 (24.8)	20 (18.1)
Higher level	568 (16.3)	521 (32)	47 (42.3)
Missing	1738 (50)		
History of smoking n (%)			
Yes	1494 (42.7)	1442 (43.3)	52 (33.8)
No	1990 (56.9)	1888 (56.7)	102 (66.2)
Missing	11(0.3)	0	11
Adult antropometry			
Body mass index (kg/m^2^)	24.8 ± 3.2	24.8 ± 3.2	24.1 ± 4.6
Lung function spirometry			
FEV1 (l per 1 s)	3.4 ± 0.7	3.4 ± 0.7	2.7 ± 0.5
FEV1 (z-score)	− 1.07 ± 1.2	− 1.07 ± 1.2	− 0.6 ± 1.2
FVC(l)	4.4 ± 0.9	4.5 ± 0.9	3.4 ± 0.6
FVC (z-score)	− 0.88 ± 1.2	− 0.88 ± 1.2	− 0.7 ± 1.1
FEV1/FVC (%)	76.9 ± 8.8	76.8 ± 8.8	81.4 ± 8.7
FEV1/FVC (z-score)	− 0.37 ± 1.3	− 0.38 ± 1.3	0.18 ± 1.4

Mean (SD) and proportions; frequency and percentage. FEV1- forced expiratory volume in one second; FVC- forced vital capacity; FEV1/FVC- ratio of FEV1 by FVC

**Table 2:** Descriptive statistics among men and women from *Malmo Offspring Study (MOS)*

	Total population (N = 1401)	Men (N = 672)	Women (N = 729)	
Age (years)	28.6 ± 6.7	28.8 ± 6.8	28.3 ± 6.5	
Birth characteristics				
Birth weight (gr)	3482.6 ± 597.9	3530.4 ± 647.2	3438.6 ± 545.3	
Birth weight (z-score)	− 0.1 ± 1.4	− 0.01 ± 1.2	− 0.18 ± 1.5	
Birth length (cm)	50.3 ± 2.4	50.8 ± 2.4	49.9 ± 2.3	
Gestational age (days)	278.3 ± 14	278.4 ± 15	278.2 ± 13.1	
Education n (%)				
Incomplete primary school	4 (0.3)	1 (0.2)	3 (0.5)	
Primary school	53 (3.8)	22 (4.1)	31 (4.9)	
Secondary school	702 (50.1)	368 (67.9)	334 (52.8)	
Higher level	415 (29.6)	151 (27.9)	264 (41.8)	
Missing	227 (16.2)			
History of smoking n (%)				
Current*	234 (16.7)	101 (18.7)	133 (20.9)	
Ex-smoker	183 (13.1)	79 (14.6)	104 (16.4)	
Never	759 (54.2)	361 (66.7)	398 (62.7)	
Missing	225 (16.1)			
Adult antropometry				
Body mass index (kg/m^2^)	24.96 ± 4.59	25.56 ± 4.41	24.40 ± 4.68	
Lung function spirometry				
FEV1 (l per 1 s)	3.6 ± 0.8	4.5 ± 0.6	3.3 ± 0.5	
FEV1 (z-score)	− 0.4 ± 0.9	− 0.5 ± 0.9	− 0.4 ± 1	
FVC(l)	4,8 ± 1.1	5.7 ± 0.8	4.0 ± 0.6	
FVC (z-score)	− 0.1 ± 0.9	− 0.1 ± 0.9	− 0.1 ± 0.9	
FEV1/FVC (%)	80.5 ± 6.8	78.7 ± 6.8	82.0 ± 0.3	
FEV1/FVC (z-score)	− 0.5 ± 0.9	− 0.6 ± 1	− 0.5 ± 0.9	

Mean (SD) and proportions; frequency and percentage. FEV1- forced expiratory volume in one second; FVC- forced vital capacity; FEV1/FVC- ratio of FEV1 by FVC. *Both regular and occasionally smoker

### Malmo Preventive Project (MPP)

Of the 3495 participants, 3330 (95.2%) were men and 165 (4.8%) women (by study design). Nearly 79% of participants had birth weights within the normal range (2500–4000 g). Of the remaining subjects, 3.5% had low birth weight (< 2500 g) and 18% had high birth weight (> 4000 g), which is representative compared to the national average for Sweden [32].

As shown in Table 1, the mean (± SD) age at MPP screening (baseline) was 46.4 (± 5.3) years for men and 49.4 (± 7.2) for women. Furthermore, women had lower adult BMI than men (p = 0.042). On the contrary, a higher percentage of men had ever smoked (44.5%) compared to women (33.8%).

### Malmo Offspring Study (MOS)

Of the 1401 participants, 672 (48%) were men and 729 (52%) women. Nearly 78% of participants were born within the normal birth weight range (2500–4000 g). Of the remaining subjects, 5.5% had low birth weight (< 2500 g) and 16.5% had high birth weight (> 4000 g). The mean (± SD) age at MOS baseline screening was 28.8 (± 6.8) years for men and 28.3 (± 6.5) for women. Of the 1401 participants, 704 (50.2%) were siblings. Additional file 1: Table S1 shows descriptive data for siblings and non-siblings. Further, as shown in Table 2, women had lower BMI than men (p < 0.001), but a higher percentage of women had ever smoked (37.3%) compared to men (33.3%). Additionally, there was a statistically significant difference in the mean z-scores of FEV_1_, FVC and FEV_1_/FVC between genders, with women showing lower spirometry values compared to men (p < 0.001) in both MPP and MOS.

### Univariate analysis

As shown in Additional file 1: Table S2, no relationship between birth parameters (birth weight and gestational age) and z-scores of adult lung function was found in MPP, while the association in MOS was very low, but significant (r < 0.1, p-value < 0.05).

### Hierarchical multiple regression analysis

Hierarchical multiple regression analyses with birth weight (z-score) as an independent variable and each z-score of adult lung function as the dependent variable are shown in Additional file 1: Table S3. In MPP, birth weight (z-score) did not associate with lung function in adult age, neither unadjusted nor in adjusted models. In contrast, in MOS participants, higher birth weight was significantly, but very weakly, associated with higher FEV_1_ (z-score) (r^2^ = 0.3%; p = 0.044), but not when adjusted for offspring’s educational level and smoking status. Also, the mixed linear model accounting for clustering showed that birth weight deviation was not a statistically significant predictor of lung function in adult life after adjustment for offspring’s educational level and smoking status (Additional file 1: Table S4).

### Logistic regression analysis

Direct logistic regression was performed to assess the impact of birth weight (z-score) on the likelihood of having impaired lung function in adult age (Table 3). In MPP, the model containing birth weight (z-score) as a predictor of lung function was non-significant, indicating that the model was not able to distinguish between offspring who had or did not have impaired lung function in adult life. However, in MOS participants birth weight was a statistically significant (p = 0.03) predictor of FEV_1_ even after adjustment for the offspring’s educational level and smoking status. Thus, for every additional unit (deviation) in birth weight, the odds of offspring having low FEV_1_ were 0.77 times less likely. Correspondingly, an increase in birth weight resulted in a decreased probability of having low FEV1, i.e. a better lung function.

**Table 3:** Logistic regression predicting adult impaired lung function among men and women in MPP and MOS

			MPP				MOS			
			Odds Ratio	95% C.I. for OR		p*	Odds Ratio	95% C.I. for OR		p*
Birth weight (z-score**)	FEV1 (z-score)	Model 0	0.981	0.926	1.039	0.515	0.783	0.668	0.919	0.003
		Model 1	0.978	0.901	1.062	0.602	0.772	0.650	0.918	0.003
	FVC (z-score)	Model 0	0.952	0.893	1.015	0.135	0.787	0.621	0.998	0.048
		Model 1	0.947	0.864	1.038	0.245	0.806	0.628	1.033	0.088
	FEV1/FVC (z-score)	Model 0	0.984	0.914	1.058	0.656	0.911	0.785	1.058	0.222
		Model 1	0.987	0.890	1.094	0.804	0.888	0.758	1.041	0.143

^*^p < 0.05 indicates statistical significance

^**^z-score value defined as less than the lower limit of normal (LLN)

Model 0—unadjusted; Model 1—adjusted for offspring’s education and smoking

OR: odds ratio; 95% C.I.: 95% confidence interval

### Two-way between-groups analysis of variance (ANOVA)

The relationship between postnatal growth patterns and lung function in adult age has been shown in Table 4.

**Table 4:** Birth weight and achieved body weight in young adult age predicting adult lung function

		MPP							MOS				
		Mean difference	Standard error	Sig (p)	Confidence interval				Mean difference	Standard error	Sig (p)	Confidence interval	
					Lower	Upper						Lower	Upper
FEV_1_ (z-score)													
Group 1 (N = 898)	Group 2 (N = 2805)	0.115	0.04	0.05	0.0001	0.230	Group 1 (N = 340)	Group 2 (N = 375)	0.05	0.07	0.87	− 0.124	0.228
Reference	Group 3 (N = 2570)	0.019	0.05	0.97	− 0.097	0.136	reference	Group 3 (N = 261)	− 0.14	0.08	0.23	− 0.336	0.05
	Group 4 (N = 701)	0.171	0.06	0.02	0.019	0.322		Group 4 (N = 306)	0.10	0.07	0.46	− 0.08	0.29
FVC (z-score)													
Group 1 (N = 899)	Group 2 (N = 2806)	0.136	0.04	0.008	0.026	0.246	Group 1 (N = 339)	Group 2 (N = 375)	− 0.08	0.07	0.60	− 0.256	0.089
Reference	Group 3 (N = 2572)	0.012	0.04	0.993	− 0.01	0.123	reference	Group 3 (N = 261)	− 0.10	0.07	0.51	− 0.291	0.088
	Group 4 (N = 703)	0.132	0.06	0.090	− 0.013	0.277		Group 4 (N = 306)	− 0.14	0.07	0.21	− 0.319	0.044
FEV_1_/FVC (z-score)													
Group 1 (N = 884)	Group 2 (N = 2778)	− 0.018	0.05	0.985	− 0.148	0.113	Group 1 (N = 339)	Group 2 (N = 375)	0.24	0.07	0.002	0.067	0.421
Reference	Group 3 (N = 2531)	0.014	0.05	0.993	− 0.118	0.146	reference	Group 3 (N = 261)	− 0.03	0.08	0.97	− 0.227	0.162
	Group 4 (N = 697)	0.074	0.07	0.678	− 0.097	0.245		Group 4 (N = 306)	0.41	0.07	< 0.001	0.223	0.595

^*^p < 0.05 indicates statistical significance; two-way ANOVA has been performed; BW: birth weight; W: weight

Group 1 = low birth weight and low weight in adult age (reference group); group 2 = high birth weight and high weight in adult age; group 3 = high birth weight and low weight in adult age; group 4 = low birth weight and high weight in adult age. Comparisons of the mean z-score were made between subgroups (1–4)

In MPP, group comparisons indicated that the mean FEV_1_ (z-score) for the *group 1* (low birth weight and low adult weight; Mean (M) − 0.98, SD 1.15) was significantly different from the means of both the *group 2* (high birth weight and high adult weight; M − 1.09, SD 1.19) and *group 4* (low birth weight and high adult weight; M − 1.15, SD 1.14), as shown in Table 4. The mean FVC differed between *group 1* (M − 0.8, SD 1.07) and *group 2* (M − 0.93, SD 1.14), while no statistically significant difference was observed for the mean FEV_1_/FVC-ratio.

In MOS, there were no statistically significant differences between groups regarding mean FEV_1_ and mean FVC (Table 4). However, the mean FEV_1_/FVC-ratio of the *group 1* (M − 0.37, SD 0.99) was significantly different from the means of both the *group 2* (high birth weight and high adult weight; M − 0.61, SD 0.88; p = 0.002) and *group 4* (low birth weight and high adult weight; M − 0.77, SD 0.9; p < 0.001), respectively.

## Discussion

In this prospective observational study, we investigated whether birth weight (z-score) is associated with impaired lung function (z-score) in adult life in two population-based cohorts, differing by age range but from the same population. Birth weight deviations were not an independent predictor of adult lung function in the elderly cohort—the Malmo Preventive Project (MPP). On the contrary, we found positive associations between birth weight and adult lung function in the younger cohort—the Malmo Offspring Study (MOS). In MOS, birth weight was a statistically significant predictor of FEV_1_ in the unadjusted model using multiple regression using continuous variable, but once we adjusted for offspring’s smoking habits and educational level, birth weight did not independently predict FEV_1_ anymore (p = 0.06). However, in the logistic regression analysis of birth weight and low FEV_1_, a category defined by a value lower than lower limits of normal (LLN), results remained statistically significant even after adjustment for offspring’s smoking and education. This indicates that offspring born with lower birth weight had 1.3 times increased odds of having a clinically important lung function impairment.

In MOS, we also performed the mixed linear model analysis to explore if birth weight can predict the lung function in adult age accounting for clustering of siblings as 50% of our participants in MOS had at least one sibling enrolled in the study. The mixed linear model analysis accounting for clustering showed that birth weight deviation was not a statistically significant predictor of lung function in adult life after adjustment for offspring’s educational level and smoking status. This finding was consistent with the results from the main hierarchical multivariate regression analysis. We also tried to perform the logistic regression (generalized linear mixed model) accounting for clustering of siblings, but due to few participants (n < 200) having impaired lung function in adult life (z-score lung value less than − 1.64), a negative Hessian matrix indicated that the model was not robust and, thus, was not reported.

Previous studies on birth weight and lung function have been conflicting, with some reporting associations [15, 18, 33–42], while others found no such associations [16, 43–49]. A systematic review by Saad et al. showed strong and consistent evidence of association between birth weight and restrictive impairment (FVC) in adulthood, but a weaker association with obstructive impairment (FEV1/FVC) [5]. A birth cohort study from Finland, performed on 5390 men and women born full term in 1966, found that FEV_1_ and FVC at the age of 31 years increased linearly with higher birth weight [34]. Moreover, a British birth cohort study that explored associations between birth weight, early growth and adult lung function at the age of 43, 53 and 60–64 years among about 3250 men and women born in 1946, found that neither FEV_1_ nor FVC were associated with birth weight in the fully adjusted models (adjustment for adult height, education, smoking status, smoking pack-years, asthma status, weight gain at age of 2 years, lower respiratory tract infection under 2 years, and childhood social class), except for FVC at the age of 53 years [18]. A limited number of studies examined this relationship when lung function was transformed into z-score. One example was a prospective cohort study from the Netherlands among 5635 children, reporting that higher birth weight was associated with higher FEV_1_ and FVC values at 10-years of age [37].

Similarly, a study from Hong Kong among 3030 children, showed that lower birth weight was associated with poorer lung function at 17.5 years of age, particularly in boys [39]. Furthermore, one case–control study from Finland that assessed lung function (z-scores) at the age of 22-years showed that young adults born with very low birth weight (< 1500 g) have reduced forced airflow regardless of a history of bronchopulmonary dysplasia [38]. However, most of these studies are limited to a follow-up during childhood, adolescence or early adulthood [10, 15, 17, 36, 39, 46, 48–50], and some studies were restricted by small sample size [43, 46, 51], or lacking data on gestational age [18, 52]. Nevertheless, the contradictory findings may even be due to heterogeneous methods applied or diverse confounders adjusted for.

Postnatal growth patterns may compensate for low birth weight and contribute to better adult lung function. Comparisons between MPP subgroups indicated that adult lung function (FEV_1_) was higher in children born with higher birth weight and who achieved a higher adult body weight (group 2), but also in children of lower birth weight achieving higher adult body weight (group 4), as compared to children with lower birth weight in combination with lower adult body weight (group 1). In addition, mean FVC was also higher in subjects with high birth weight achieving a higher adult body weight (group 2) compared to babies born with lower weight who stayed within a lower adult body weight (group 1). These findings might be explained by anatomical aspects of wider bronchi and increased lung volumes in people with larger bodies.

However, in MOS, no difference in mean FEV_1_ or FVC between these sub-groups were found. Nevertheless, mean FEV_1_/FVC-ratio was higher in adults born with lower birth weight achieving higher adult weight (group 4) compared to those born with low weight who stayed within the low adult weight category (group 1).

Body mass index and adiposity growth during childhood for predicting young adult asthma has been studied previously [53]. The mis-match concept was first developed to address increased cardiometabolic risk in babies born small and achieving a high childhood or adult BMI [54, 55], but also according to changes in kidney function [56] and the increased risk of respiratory disease [51], most notably a decrease of FEV in mis-match young babies at age 4–15 weeks [57]. Our findings, on the contrary, showed that small babies growing big (> 74 kg) in early adult life have a better adult lung function than small babies achieving lower adult weight. Hence, not just the birth weight, but even the postnatal growth pattern seems to be of importance, because low birth weight can be compensated for if you are allowed to grow big, at least for lung function. This is partly consistent with findings from an Australian study that explored the impact of catch-up growth and childhood BMI on lung function at the age of 21 years showing a positive contribution of catch-up growth and increasing BMI at the age of 5 years to better lung function [55]. Our findings are further supported by another recent study by Voraphani et al., who used data from three population-based birth cohorts (one from Sweden) to identify early life risk factors for spirometric restriction in adult life and showed associations between being born small for gestational age and being underweight in childhood and spirometric restriction in adulthood (up to 36 years of age) [58]. However, if weight gain or onset of obesity occurred after 5 years of age, an adverse impact on adult lung function was noted [50].

The discrepancy in our findings between MPP and MOS may be explained by cohort differences that may reflect selection bias or birth cohort effect [59]. Differences may also exist between generations, not just due to chronological age, but even the historical development in Sweden, as well as lifestyle habits. Therefore, we did not pool the data from MPP and MOS, but instead have performed analyses separately. For instance, preventive maternal health care was introduced in 1947 in Sweden on a national scale (and further improved in 1955), which might have affected lifestyle and medical care of mothers to individuals in MOS but not in MPP. This implies that preventive maternal health care could play an important role for fetal health with resulting improved birth outcomes. Moreover, different generations of spirometry devices have been used in these cohorts with baseline examinations more than 25 years apart.

Limitations of the present study include the lack of information on maternal smoking during pregnancy, as well as data on lung diseases in childhood. Also, we have been restricted by a small sample size in the final analysis due to missing data on birth parameters (birth weight and gestational age) and spirometry results for more than half of the population from the original cohort. The degree of attendance in MOS in general is 47%. MOS consists of children and grandchildren to the index-participants in the MDCS Cardiovascular Arm with 47% attendance rate in general [49]. Birth data and other perinatal data are derived from the Swedish Medical Birth Register (MBR) which covers all births in Sweden since 1973 on a national scale. For a total of 1401 subjects in MOS born in 1973 or later, birth weight data was available in MBR and only those were therefore included. Birth data was regretfully not available for MOS participants born before 1973. Also, birth data of men in MPP was available for participants who were originally enrolled in a case–control study that investigated the risk of prostate cancer, which entails potential design and selection bias. However, prostate cancer occurs much later in life, normally during the 6th or 7th decade, in relation to the age (mean 46 years) at which spirometry was performed and should thereby not have influenced the lung function test. Moreover, there is a predominance of men in MPP which reflects that 2/3 of all attendees at baseline were men, as the initial design of the MPP study was to find high-risk individuals in mid-life for preventive intervention on cardiovascular risk factors, alcohol abuse and impaired glucose tolerance, why initially only men were screened [19]. Later, when mammography screening had been implemented in Sweden in the late 1970’ ies, the idea to find high-risk individuals for preventive interventions also in women was proposed. Therefore, women were invited to attend the screening program as further described [19], which is the reason why women unfortunately are underrepresented (1/3 of all subjects in MPP). We consider that women should be included in the analysis as it is of great importance to have both genders represented in a study on lung function because gender differences may exist. Women in general have differing anatomical features as compared to men (thorax size, bronchial diameter, etc.) and, in addition, may have experienced different life course trajectories (occupational exposures, smoking habits) than men. In addition, including women contributes to the statistical power of the study. Moreover, we have performed multiple tests and are aware that this may be a concern as we not only have three outcome variables (FEV1, FVC and FEV1/FVC-ratio) but even two cohorts. This may increase the risk of chance findings, however, we are very cautious in interpretation of our results because of this. A final limitation is the lack of clinical endpoints. This is because only a few subjects have so far been hospitalised for lung disease in MOS. Most patients with lung problems are diagnosed, treated and followed in primary health care, but unfortunately no national registers cover this.

The major strengths of this study include the cohort study design and the long follow-up time. Lung function was assessed at a mean age of 46 and 28 years in MPP and MOS, respectively. Moreover, these cohorts offer objective birth register-based data, as well as information on gestational age based on medical archives or national registers. Until now, there are few cohorts providing information on both objective birth weight adjusted for gestational age and adult lung function test. An additional important strength is that we analyzed lung function measurements transformed into z-scores. Absolute lung function measurements reflect lung size which depends on several factors, such as body size, dimensions of the thoracic cavity, sex and age. The z-score takes into account age, sex, height and ethnic group and thereby minimizes the age- and height-related bias, thus enabling the comparison of lung function between individuals, irrespective of their age, sex, height, or ethnicity [30]. Finally, we consider that our results represent an underestimation of true associations as a number of measurement variations and imprecisions may affect epidemiological studies based on technical measurements carried out over longer time periods (several years) and thereby dilute associations.

## Conclusions

In summary, we report that babies born with lower birth weight, adjusted for gestational age, are more likely to have impaired lung function in adult life—a finding more visible in a younger and more recent birth cohort. This association is relatively weak and could have been taken over by other factors acting in adult life, such as smoking and educational level, closer to the spirometry examination. A difference may exist between generations, not just because of the time interval, but because of lifestyle changes (smoking habits) and historical developments in Sweden. Finally, we would like to underline the importance of postnatal growth patterns that may compensate for low birth weight and contribute to better adult lung function. Such a mis-match seems to be of benefit for adult lung function, as contrary to the increased risk of adult cardiometabolic disorders [54, 55]. The most likely reason is the anatomy of the thorax, bronchi and lung function in relation to body size [60, 61].

The clinical application of the findings is not immediate as this is not a clinical study per se involving patients, but a population-based epidemiological study. However, the data add to a growing interest in clinical lung medicine to better understand the early life origins and modifiers of adult lung function. This could increase the interest in early prevention of lung disorders when healthy pregnancies and normal birth outcomes could be of importance, as well as healthy weight trajectories in childhood and adolescence.

## Supplementary Information

**Additional file 1: Table S1.** Descriptive statistics among MOS *siblings* and *non-siblings.* **Table S2.** Associations between birth parameters and lung function in MPP and MOS. **Table S3.** Birth weight for prediction of lung function in MPP and MOS. **Table S4.** Birth weight for prediction of lung function accounting for MOS siblings.

**Additional file 2: Figure S1.** Flowchart of the Malmo Offspring Study (MOS) population. **Figure S2.** Flowchart of the Malmo Prospective Study (MPP) population.

## Data Availability

The dataset used for the current study are available from the Board of the Malmö cohorts (chair: Professor Olle Melander, mail: olle.melander@med.lu.se) upon reasonable request, see link: https://www.malmo-kohorter.lu.se/malmo-cohorts **Ethics approval and consent to participate** Ethical clearance was granted for the MPP study (Dnr. 2004/85) and MOS study (Dnr. 2012/594) by the Regional Ethics Board in Lund.

## Abbreviations

BW: Birth weight
MPP: Malmo Preventive Project
MOS: Malmo Offspring Study
DoHAD: Developmental Origins of Health and Disease
PAR: Predictive adaptive response
IUGR: Intrauterine growth restriction
SGA: Small for gestational age: BMI: body mass index
FEV1: Forced expiratory volume in one second
FVC: Forced vital capacity
FEV1/FVC: Ratio of FEV1 by FVC
LLN: Lower limit of normal
GLI: Global Lung Function Initiative
DAG: Directed Acyclical Graph
C.I.: Confidence interval
S.E.: Standard error
SD: Standard deviation
df: Degree of freedom
