# Supplementary material for: Can birth weight predict offspring’s lung function in adult age? Evidence from two Swedish birth cohorts
Source: Respir Res. 2022 Dec 15;23:348. doi: 10.1186/s12931-022-02269-2 (PMC9753232; doi:10.1186/s12931-022-02269-2)
Supplement: Supplementary file 1 — Additional file 1: Table S1. Descriptive statistics among MOS siblings and non-siblings. Table S2. Associations between birth parameters and lung function in MPP and MOS. Table S3. Birth weight for prediction of lung function in MPP and MOS. Table S4. Birth weight for prediction of lung function accounting for MOS siblings. [file 12931_2022_2269_MOESM1_ESM.pdf]

Additional file 1

Supplementary Table 1. Descriptive statistics among MOS *siblings* and *non-siblings*.

	**Siblings** (N= 704)	**Non-siblings** (N=694)
**SEX**		
Men (N)	329 (46.7%)	343 (49.4%)
Women (N)	375 (53.3%)	351 (59.6%)
**ADULT ANTROPOMETRY**		
Age (years)	27.9 ± 5.6	29.3 ± 7.4
Height (cm)	174.5 ± 9.7	174.9 ± 9.5
BMI (kg/m^2)	24.9 ± 4.7	25 ± 4.4
**BIRTH CHARACTERISTICS**		
Birth weight (gram)	3449.9 ± 614.3	3516 ± 580.7
Birth weight (z-score)	-0.1 ±1.6	-0.1 ± 1.2
Gestational age (weeks)	39.2 ± 2.2	39.5 ± 1.9
Birth length (cm)	50.2 ± 2.2	50.4 ± 2.5
**EDUCATION**		
Primary school	24 (3.4%)	29 (4.2%)
Secondary school	365 (51.8%)	337 (48.6%)
Higher level	216 (30.7%)	199 (28.7%)
Missing	96 (14.1%)	128 (18.5%)
**SMOKING HISTORY**		
Yes*	117 (16.6%)	117 (16.9%)
No**	494 (70.2%)	448 (64.4%)
Missing	93 (13.2%)	129 (18.7%)
**LUNG FUNCTION**		
FEV1 (l per 1 sec)	3.9 ± 0.8	3.8 ± 0.8
FEV1 (z-score)	-0.4 ± 0.9	-0.4 ± 1
FVC(l)	4.8 ± 1.1	4.8 ± 1
FVC (z-score)	-0.1 ± 0.9	-0.1 ± 1
FEV1/FVC (%)	80.9 ± 6.7	80.1 ± 6.9
FEV1/FVC (z-score)	-0.5 ± 0.9	-0.6 ± 0.9

Mean (SD) and proportions; frequency and percentage. FEV1- forced expiratory volume in one second; FVC- forced vital capacity; FEV1/FVC- ratio of FEV1 by FVC.

Supplementary Table 2. Associations between birth parameters and lung function in MPP and MOS.

	MPP			MOS		
	**FEV1 (z-score) r (p)**	**FVC (z-score) r (p)**	**FEV1/FVC (z-score) r (p)**	**FEV1 (z-score) r (p)**	**FVC (z-score) r (p)**	**FEV1/FVC (z-score) r (p)**
**Birth weight (gram)**	0.013 (0.449)	0.005 (0.763)	0.010 (0.573)	0.082 ***(0.003)***	0.038 *(0.174)*	0.057 ***(0.04)***
**Gestational age (days)**	-0.016 (0.363)	-0.017 (0.325)	-0.007 (0.691)	0.07 ***(0.012)***	0.018 *(0.514)*	0.079 ***(0.005)***
**Birth weight (z-score)**	0.019 (0.289)	0.018 (0.304)	0.004 (0.829)	0.067 ***(0.017)***	0.053 *(0.059)*	0.01 *(0.712)*
**Birth length (cm)**	-0.015 (0.387)	-0.020 (0.251)	0.003 (0.853)	0.029 *(0.312)*	0.022 *(0.431)*	0.005 *(0.872)*

Univariate analyses; r= correlation coefficient; p= p-value; * p< 0.05 indicates statistical significance

Supplementary Table 3. Birth weight for prediction of lung function in MPP and MOS.

		MPP				MOS			
		**R**	**R^2**	**95% CI**	**p-value**	**R**	**R^2**	**95% CI**	**p-value**
**FEV1 (z-score)**	**Model 0**	0.019	0.000	-0.014 – 0.048	0.290	0.057	0.003	0 - 0.08	**0.044**
	**Model 1**	0.071	0.005	-0.01 – 0.051	0.477	0.07	0.005	0 - 0.09	0.059
	**Model 2**	0.226	0.051	-0.021 – 0.062	0.362	0.072	0.005	0 - 0.09	0.062
**FVC (z-score)**	**Model 0**	0.013	0.000	-0.014 – 0.046	0.456	0.051	0.003	0 - 0.08	0.07
	**Model 1**	0.071	0.005	-0.025 – 0.057	0.612	0.065	0.004	-0.01 - 0.08	0.092
	**Model 2**	0.180	0.032	-0.022 – 0.059	0.555	0.089	0.008	-0.01 - 0.08	0.098
**FEV1/FVC (z-score)**	**Model 0**	0.006	0.000	-0.031 – 0.039	0.729	0.033	0.005	-0.02 - 0.07	0.234
	**Model 1**	0.019	0.000	-0.044 – 0.051	0.811	0.058	0.021	-0.02 - 0.07	0.261
	**Model 2**	0.107	0.011	-0.042 – 0.053	0.745	0.076	0.006	-0.02 - 0.07	0.323

Model 0: unadjusted model; **Model 1:** adjusted for offspring's education; **Model 2:** adjusted for offspring's education and smoking history. * p<0.05 indicates statistical significance; birth weight transformed to z-score; FEV1, FVC and FEV1/FVC-ratio transformed to z-score.

Supplementary Table 4. Birth weight for prediction of lung function accounting for MOS siblings.

		Estimate	CI 95%	p-value
**FEV1 (z-score)**	**Model 0**	0.019	-0.02 - 0.06	0.329
	**Model 1**	0.022	-0.02 - 0.06	0.266
	**Model 2**	0.022	-0.02 - 0.06	0.276
**FVC (z-score)**	**Model 0**	0.01	-0.03 - 0.05	0.580
	**Model 1**	0.011	-0.03 - 0.05	0.562
	**Model 2**	0.012	-0.03 - 0.05	0.556
**FEV1/FVC (z-score)**	**Model 0**	0.002	-0.04 - 0.04	0.904
	**Model 1**	0.006	-0.03 - 0.05	0.753
	**Model 2**	0.006	-0.04 - 0.05	0.780

Model 0: unadjusted model; **Model 1**: adjusted for offspring's education; **Model 2:** adjusted for offspring's education and smoking history; * $p<0.05$ indicates statistical significance; birth weight transformed to z-score; FEV1, FVC and FEV1/FVC-ratio transformed to z-score.
