# Supplementary material for: Can birth weight predict offspring’s lung function in adult age? Evidence from two Swedish birth cohorts
Source: Respir Res. 2022 Dec 15;23:348. doi: 10.1186/s12931-022-02269-2 (PMC9753232; doi:10.1186/s12931-022-02269-2)
Supplement: Supplementary file 2 — Additional file 2: Figure S1. Flowchart of the Malmo Offspring Study (MOS) population. Figure S2. Flowchart of the Malmo Prospective Study (MPP) population. [file 12931_2022_2269_MOESM2_ESM.docx]

**Additional file 2**

**Supplementary Figure 1.** Flowchart of the Malmo Prospective Study (MPP) population.

Totally **3 495** (men 3 330; women 165) participants were included in the study having available data on both birth weight and lung function

N= **28 934** underwent spirometry

(men 21 180; women 7 748)

**Baseline MPP examination**

N=**33 346**

(men 22 444; women 10 902)

N=**4 359** with available perinatal data

(men 3 883; women 476)

**Supplementary Figure 2.** Flowchart of the Malmo Offspring Study (MOS) population.

N= **28 098** participants in the Malmö Diet Cancer Study (MDCS)

N=**6 103** participants from MDCS who participated in MDCS-Cardiovascular Cohort (MDCS-CC)

N= **3 200** attending MOS out of potential 10,202 adult children and grandchildren to MDCS-CC participants

N = **1 401** participants born 1973 and later included in the present study with available data on birth weight from Swedish Medical Birth Register (MBR) starting in 1973
